# Assessment of NER solutions against the first and second CALBC Silver Standard Corpus

**DOI:** 10.1186/2041-1480-2-S5-S11

**Published:** 2011-10-06

**Authors:** Dietrich Rebholz-Schuhmann, Antonio Jimeno Yepes, Chen Li, Senay Kafkas, Ian Lewin, Ning Kang, Peter Corbett, David Milward, Ekaterina Buyko, Elena Beisswanger, Kerstin Hornbostel, Alexandre Kouznetsov, René Witte, Jonas B Laurila, Christopher JO Baker, Cheng-Ju Kuo, Simone Clematide, Fabio Rinaldi, Richárd Farkas, György Móra, Kazuo Hara, Laura I Furlong, Michael Rautschka, Mariana Lara Neves, Alberto Pascual-Montano, Qi Wei, Nigel Collier, Md Faisal Mahbub Chowdhury, Alberto Lavelli, Rafael Berlanga, Roser Morante, Vincent Van Asch, Walter Daelemans, José Luís Marina, Erik van Mulligen, Jan Kors, Udo Hahn

**Affiliations:** 1 EMBL Outstation, European Bioinformatics Institute, Hinxton, Cambridge, CB10 1SD, U.K; 2 Dept. of Medical Informatics, Erasmus University Medical Center, Rotterdam, NL; 3 Linguamatics Ltd, St. John's Innovation Centre, Cambridge, U.K; 4 Language & Information Engineering (JULIE) Lab, Friedrich-Schiller-Universität, Jena, Germany; 5 Dept. of Computer Science & Applied Statistics, University of New Brunswick, Canada; 6 Dept. of Computer Science & Software Engineering, Concordia University, Montreal, Canada; 7 Institute of Information Science, Academia Sinica, Taipei 115, Taiwan; 8 University of Zürich, Switzerland; 9 Research Group on Artificial Intelligence, Hungarian Academy of Sciences, Hungary; 10 Nara Institute of Science and Technology, Nara, Japan; 11 Research Programme on Biomedical Informatics (GRIB), IMIM (Hospital del Mar Research Institute), Universitat Pompeu Fabra, Barcelona, Spain; 12 National Center for Biotechnology-CSIC, Madrid, Spain; 13 National Institute of Informatics, Tokyo, Japan; 14 Fondazione Bruno Kessler, Trento, Italy; 15 Universitat Jaume I, Spain; 16 CLiPS, University of Antwerp, Belgium; 17 Complutense University of Madrid, Spain

## Abstract

**Background:**

Competitions in text mining have been used to measure the performance of automatic text processing solutions against a manually annotated gold standard corpus (GSC). The preparation of the GSC is time-consuming and costly and the final corpus consists at the most of a few thousand documents annotated with a limited set of semantic groups. To overcome these shortcomings, the CALBC project partners (PPs) have produced a large-scale annotated biomedical corpus with four different semantic groups through the harmonisation of annotations from automatic text mining solutions, the first version of the Silver Standard Corpus (SSC-I). The four semantic groups are chemical entities and drugs (CHED), genes and proteins (PRGE), diseases and disorders (DISO) and species (SPE). This corpus has been used for the First CALBC Challenge asking the participants to annotate the corpus with their text processing solutions.

**Results:**

All four PPs from the CALBC project and in addition, 12 challenge participants (CPs) contributed annotated data sets for an evaluation against the SSC-I. CPs could ignore the training data and deliver the annotations from their genuine annotation system, or could train a machine-learning approach on the provided pre-annotated data. In general, the performances of the annotation solutions were lower for entities from the categories CHED and PRGE in comparison to the identification of entities categorized as DISO and SPE. The best performance over all semantic groups were achieved from two annotation solutions that have been trained on the SSC-I.

The data sets from participants were used to generate the harmonised Silver Standard Corpus II (SSC-II), if the participant did not make use of the annotated data set from the SSC-I for training purposes. The performances of the participants’ solutions were again measured against the SSC-II. The performances of the annotation solutions showed again better results for DISO and SPE in comparison to CHED and PRGE.

**Conclusions:**

The SSC-I delivers a large set of annotations (1,121,705) for a large number of documents (100,000 Medline abstracts). The annotations cover four different semantic groups and are sufficiently homogeneous to be reproduced with a trained classifier leading to an average F-measure of 85%. Benchmarking the annotation solutions against the SSC-II leads to better performance for the CPs’ annotation solutions in comparison to the SSC-I.

## Background

Biomedical text mining (TM) has developed into a bioinformatics discipline leading to the development IT methods that deliver accurate results from an automatic literature analysis into bioinformatics research. This research work requires the development of benchmark data sets containing annotations and thereafter the assessment of existing TM solutions against these corpora. A number of challenges have been proposed to achieve this goal: BioCreAtive I and II, JNLPBA and others [1-5]. In all these approaches, the organisers deliver a set of manually annotated documents and ask the challenge participants (CPs) to reproduce the results with their automatic methods. The annotated corpora are provided to the public after the challenge is closed and all the results are documented and published in a scientific manuscript.

The first CALBC Challenge is similar in the sense that the project partners (PPs) of the CALBC project also provided an annotated corpus to the CPs of the first CALBC challenge to reproduce the annotations with automatic means. On the other side, the first CALBC Challenge was different to the before-mentioned challenges with regards to the following modifications: (1) the annotated corpus has been generated automatically and not manually (Silver Standard Corpus, SSC-I), and (2) the size of the SSC-I is significantly bigger than the corpora mentioned produced for the other challenges, i.e. the annotated corpus contains 50,000 Medline abstracts for training and the corpus for annotation consists of 100,000 test documents. This difference in size requires that all assessment is performed fully automatically, that the CPs apply annotation solutions that can cope with such a large-scale corpus and that the assessment solutions can evaluate the contributions in a short period of time. The automatic annotation of the corpus also requires new solutions to integrate the contributions from different automatic annotation solutions into a single corpus. This process will be called “harmonisation” and refers to methods that measure the agreement between the boundaries from different annotation solutions to filter out entity boundaries that fulfil consensus criteria. Overall these annotations should have the characteristic that all annotation solutions show high performance against the set of annotations, for example when measuring the F-measure of the annotation solution [6].

When comparing different NER solutions, it becomes clear that they do not generate the same results depending on their approach, their implementation, and the type of resources used for the instantiation of the solutions (see BioCreative II). On the other side, when combining the results from different automatic annotation solutions, we can achieve an improvement of the results of the combined solution (see BioCreative Meta-Server) [7]. As a consequence, the PPs of the CALBC project have combined their automatic annotation solutions to produce the first Silver Standard Corpus of the CALBC project [8].

In addition, each annotation solution is optimised for a single semantic type and solutions for a larger scope of semantic groups are still missing. This is again partly due to the fact that manually curated corpora can only cover a small number of semantic groups to focus the ongoing work to the amount of work that is achievable in a fixed period of time and according to the available budget. The proposed approach of the CALBC project can cover a larger number of annotations due to the fact that the annotations are produced automatically and harmonised with automatic means.

In this manuscript, we report on the results of the first CALBC challenge. The CPs have submitted one or several sets of annotated documents. All the submissions have been assessed against the SSC-I. In addition, the submissions have been used to generate the second Silver Standard Corpus (SSC-II) and all the submissions have been assessed against the SSC-II. The results are presented in this manuscript to support a better understanding to which extent the automatic generation of an annotated corpus contributes to the benchmarking of annotation solutions in a domain where a large number of NERs have to be identified inside a large number of scientific documents.

## Methods

In the CALBC project and challenge the PPs and CPs contribute their annotations on a given corpus to enable the harmonisation of all annotations for a large-scale annotated corpus. A priori we can assume that the annotation solutions do not share any properties and the contributed annotations should be produced by independent systems, but should be similar in the sense that they contribute annotations for entities in the biomedical domain. This leads to the result that the different solutions make use of similar biomedical data resources for the representation of terms and concepts and thus are expected to show similarities in the annotation.

### Generation of the first CALBC Silver Standard Corpus (SSC-I)

All PPs annotated the corpus of 150,000 Medline abstracts with their annotation solutions. The project partners P01, P02 and P04 used dictionary-based concept recognition methods with techniques for quality improvements, whereas partner P03 applied a combination of solutions that are either dictionary-based or is based on machine-learning techniques. All annotations were delivered in the IeXML format and concept normalisation should make use of standard resources such as UMLS, UniProtKb, EntrezGene or should follow the UMLS semantic type system [9-13].

The alignment is based on the methods described in [6,14]. The applied method used pair-wise comparisons of annotated sentences considering all tokens and their order (called “alignment”) between the two sets from two different sources for a given semantic type. For every sentence the annotations from one contribution for a given type is aligned with the annotations from the next contribution for the same semantic type. The tokens have been weighted with the inverse document frequency (IDF) for the tokens across the whole corpus and the cosine similarity of the two annotations has been measured. If the similarity is above 0.98, then the alignment is considered successful and the boundaries of the shorter annotation have been selected as the final annotation (called “harmonisation step”). If the contributions from at least two partner agree on the same annotation (2-vote agreement), then the annotation has been selected for the final corpus. Only in the case of entities belonging to the category CHED, the PPs shared the identical representation of a terminological resource for the annotation task but this did not lead to a higher agreement on the annotations than for the other categories [15].

### Generation of the SSC-II

The contributions of the CPs were evaluated against the SSC-I. Different evaluation schemes were used to determine the performance of the solutions [6,14]. All contributions were assessed against the SSC-I by applying exact matching, nested matching and cosine similarity matching with a 0.98 and 0.9 cosine similarity score (results not shown). The measurements were performed on the basis of a set of 1,000 Medline abstracts that have been selected at random from the full corpus.

The best average F-measure performances were achieved when applying 0.9 and 0.98 cosine similarity scoring. All submissions from all participants have been evaluated and the contributions with the best F-measure performance against the SSC-I have been selected for the harmonisation methods into the SSC-II. For the harmonisation of the contributions (SSC-II), a varying number of contributions had to be considered for the different semantic groups, i.e. 6 for CHED, 7 for SPE, 8 for DISO and 9 for PRGE (ref. to table 1). A 3-vote agreement in combination with a 0.98 cosine similarity score in the alignment was required for the acceptance of the annotation across the different contributions. For all semantic groups, different voting schemes (i.e. 2- to 6-vote agreement) were evaluated to determine the best performing voting scheme in terms of the highest average F-measure performance across the contributions of the CPs. The 3-vote agreement delivered the best balance between the recall and the precision of the contributions against the harmonised SSC-II. All presented results are based on the annotations on the subset of 1,000 Medline abstracts.

**Table 1:** The table gives an overview on the annotation solutions that have been used for the generation of the SSC-I and the SSC-II. For the generation of the SSC-I only the annotations from the 4 project partners (P01 – P04) have been integrated, whereas the SSC-II combines the annotations from the challenge participants (P06-P10, P13 and P15), not including P11, P12 and P14, since they have used the training data. Please refer to the proceedings of the first CALBC workshop for further details [8].

Solution	PPs | CPs	Use of Training Data	PRGE	CHED	DISO	SPE
Dictionary-based concept recognition	P01	[ / ]	UniProtKb	Jochem	UMLS	NCBI taxonomy
	P02	[ / ]	Different resources incl. UniProtKb, EntrezGene	Jochem	UMLS	NCBI taxonomy
	P04	[ / ]	UniProtKb, EntrezGene	Jochem	MeSH, MedDRA, NCI, SNOMED-CT UMLS	NCI, MeSH, SNOMED-CT
	P06	[ / ]				
	P10	[ / ]	UniProtKb, EntrezGene			NCBI taxonomy
	P13	[ / ]				

Indexing of tokens and terms	P15	[ / ]	UMLS	UMLS	UMLS	UMLS

Both, trained & rule-based solutions	P03	[ / ]	UniProtKb, EntrezGene	Jochem	UMLS	NCBI taxonomy

Case-based reasoning	P09	[ / ]			UMLS	

CRF based, trained NER solution	P07	[ / ]				
	P16	[ / ]	Genia		UMLS	
	
	P11	YES	[ / ]	[ / ]	[ / ]	[ / ]
	P12	YES	[ / ]	[ / ]	[ / ]	[ / ]
	P14	YES	[ / ]	[ / ]	[ / ]	[ / ]

The alignments of the 100,000 documents were either performed on Sun Fire opteron servers (4 or 8 CPUs, RAM sizes from 32 to 256 Gb RAM, 9-12 hours) or on the compute farm of 700 IBM compute engines (dual CPU, 1.2-2.8 Ghz, 2 GB RAM, 3 hours).

### Challenge participation and challenge contributions

12 CPs took actively part in the challenge. Each CP could contribute several submissions at any time. Overall the CALBC challenge received 19 valid submissions. 3 CPs used the SSC-I as training data and contributed in total 8 submissions (ref. to table 2). 2 CPs did not use the SSC-I, but used an annotation solution that has been trained on a different annotated corpus for the challenge. All other partners (in total 11) used dictionary-based solutions and in one case used a combination of different solutions without training on the SSC-I.

**Table 2:** The table shows the number of annotations that are contained in the SSC-I. This corpus has been generated from the contributions of the PPs. Not all challenge participants (CPs) have participated in all parts of the challenge. A smaller number of CPs has submitted annotations for chemical entities. The average number of annotations for CHED and PRGE in the submitted corpora was above the number of annotations in the SSC-I and for DISO and SPE below the number of the ones in the SSC-I.

	Nr. of annotations in SSC-I	Nr. of CPs	Nr. of Submissions from CPs	Average nr. of annotations from all CPs	Nr. of annotations in SSC-II
CHED	228,622	6	11	233,398	238,431
PRGE	275,235	9	15	343,681	435,797

DISO	300,637	8	11	255,599	245,524
SPE	317,211	7	9	277,071	304,503

Five CPs only focused on a single semantic group. All the other CPs covered three or more semantic groups. CP P10 delivered for PRGE a very high number of annotations, which impaired the performance of the system against the SSC-I.

## Results

The PPs contributions have been aligned to generate the SSC-I. The SSC-I has been contributed to the public to train machine-learning based NER solutions on the corpus and to gather the annotations of the CPs for performance assessments. The contributions of the CPs have been used to generate the SSC-II.

### Evaluation of the contributed annotated corpora against the SSC-I

The submissions of the CPs were compared against the SSC-I (see below). Table 3 shows that two solutions that were based on the provided training data reproduced the SSC-I annotation „standard“ at a high level of quality: for SPE the solutions achieved 93% F-measure and for the other semantic groups the F-measure was above 80% [8]. This shows that the SSC-I was homogeneous enough so that a trained system could reproduce the annotations. As a consequence, we can expect that complex automatic annotation solutions could be replaced with a machine learning approach to reproduce the annotations.

**Table 3:** The table shows the F-measure performance of the PPs and the CPs against the SSC-I (cos-98 harmonisation, 2 vote agreement). The project partners are part of the comparison (P01 – P04). P11, P12, and P14 used the training data for their annotations. Only the best performing submission of each CP was included into the analysis. P09 only contributed a small number of annotations in the submitted corpus.

Cos-98	***P12***	***P11***	P03	P04	P01	P02	P10	***P14***	P08	P15	P09	P06	P07	P09	P13	P16
SPE	93%	93%	79%	83%	71%	69%	84%	69%	56%	42%	2%					
DISO	87%	89%	71%	69%	82%	76%	78%	62%	51%	32%	3%	73%				

CHEM	**83%**	**84%**	75%	82%	49%	68%		51%	20%	17%	3%				23%	
PRGE	**81%**	**73%**	77%	66%	66%	59%	40%	52%	12%	18%	2%		50%	11%		28%

Avg.	86%	85%	76%	75%	67%	68%	68%	58%	35%	27%	2%					

### Performance measures of the CPs’ solutions against the SSC-I

The performances of the annotation solutions against the SSC-I and the SSC-II .

**Figure 1:**
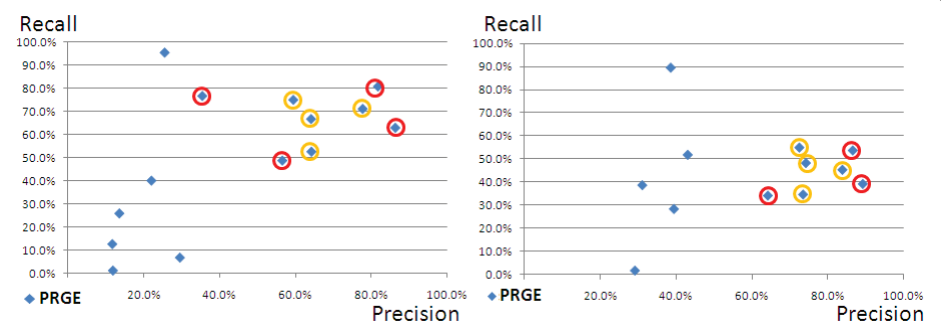
**(Proteins/Genes in SSC-I and SSC-II):** The figure displays the comparison of the CPs performances to the SSC-I (left side) and to the SSC-II (right part) for the annotation of proteins and genes (PRGE). Only a restricted number of annotated copora from CPs can be measured against the SSC-II, since a few submissions are based on solutions that have been trained on the SSC-I. The two diagrams display scatter plots for the precision and recall values of the different annotation solutions. Red circles denote systems that have used the training data and yellow circles denote the annotations delivered by the PPs’ annotation solutions.

The PPs’ tagging solutions share the same range of performance, i.e. their precision and their recall ranges from 55 to 80% (fig. 1). The performance of CPs’ solutions that did not rely on the training data was lower than the PPs’ performances. Two of the trained systems showed higher performances than the PPs’ solutions.

The two best-performing machine-learning based solutions produce results that are comparable to known solutions for the gene mention task [16,17]. On the other side, the performances have been measured against a corpus that includes a higher degree of variability in the annotations in comparison to the gold standard corpora that are usually used for the measurement of gene-tagging solutions.

**Figure 2:**
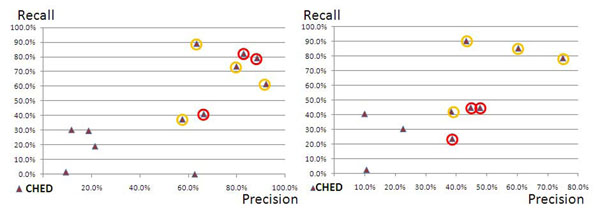
**(Chemical Entities in SSC-I and SSC-II):** The figure displays the performances for the PPs’ and CPs’ annotated datasets for chemical entities (CHED) measured against the SSC-I (left side) and the SSC-II (right part). For further details please refer to Fig. 1.

Fig. 2 shows a distribution for the performance for the annotation for chemical entities. The two best performing machine-learning solutions outperform again all other annotation solutions and the PPs’ annotation solutions have performances that are rather similar to each other and quite different from the performances of the contributions from the CPs. Comparing the results in fig. 1 and fig. 2, we note that the best annotation solutions show better performance for chemical entities than the same solutions demonstrate for the annotation of proteins/genes. We conclude that the annotation of PRGEs in the SSC-I have higher variability (or more noise) than the annotations for the chemical entities in the same corpora.

**Figure 3:**
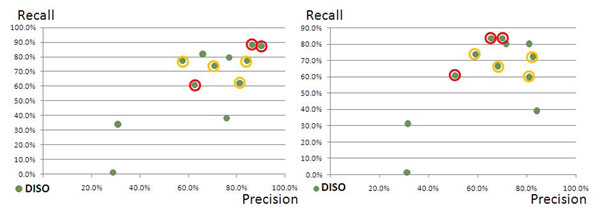
**(Diseases in SSC-I and SSC-II):** Distribution of the CPs’ contributions in a Prec/Rec scatter plot for the category of disease annotations (DISO). The best performing solutions were again trained on the training data and achieved performances of almost 90% recall at 90% precision.

**Figure 4:**
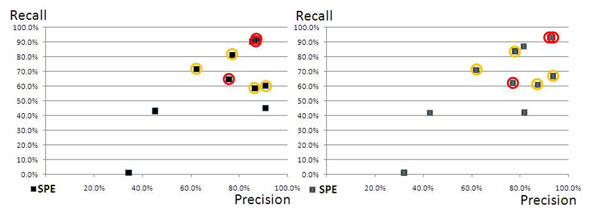
**(Species in SSC-I and SSC-II):** Distribution of the CPs’ contributions in a Prec/Rec scatter plot for species (SPE). The two machine-learning approaches showed almost identical performances.

The following figures (fig. 3 and 4) show the same distribution for disease and species mention identification. For these two tasks the annotation solutions show better performance than for the previous two tasks (PRGE and CHED). We can derive that a good performance on these two tasks can be reached by the majority of the annotation solutions in comparison to the other two tasks.

The diagram for the identification of the diseases (DISO, fig. 3) demonstrates that the majority of the proposed systems identified the diseases at a recall of 60% and above, and at a precision of 55% and above. Two rule-based solutions from CPs showed similar performances to the PP’s solutions. We can conclude that the representation of the diseases in the SSC-I is better standardised and thus includes less variability or noise than the representation of proteins/genes and chemical entities.

The identification of species could be solved to the best precision and the best recall values from the large majority of all proposed solutions. Again the two best performances were achieved by two machine-learning approaches that reproduced the annotations from the training data. The performances of the other solutions, i.e. the PPs’ solutions and the CPs’ solutions, had the best performances for the identification of species in contrast to the other tasks. It is clear that the identification of species can be performed at a level of quality which is above the measured performances of the other semantic groups.

### Performance against the SSC-II

The results of the CPs and the PPs were compared against the SSC-II in addition to the SSC-I. Both harmonised sets were generated by using 98% cosine similarity and the comparison against the corpus was done with the same measure (ref. to table 3 and 4).

**Table 4:** F-measure performance of the contributions from the PPs and the challenge participants against the SSC-II (harmonisation: 98% cosine similarity, 3 vote agreement, 1,030 documents, see Material & Methods).

	Partners				Participants						
	P01	P02	P03	P04	P08	P09	P15	P06	P10	P16	avg
SPE	69.9%	66.6%	72.6%	79.1%	60.2%	2.3%	44.2%		87.8%		60.3%
DISO	77.2%	67.4%	68.9%	65.6%	53.5%	2.5%	31.5%	75.7%	80.6%		58.1%

CHEM	40.3%	76.8%	70.8%	58.6%	26.0%	4.4%	16.0%				41.8%
PRGE	62.6%	47.1%	58.9%	58.6%	33.1%	3.2%	34.6%		54.0%	47.1%	44.4%

### Tagging of proteins/genes and chemical entities measured against the SSC-II

The performances of the PPs’ annotation solutions for genes/proteins showed lower results in the assessment against the SSC-II than in comparison to the SSC-I (ref. to fig. 1). Since the SSC-II represents the harmonisation of annotations across a larger number of contributions, it can be expected that the annotations in the SSC-II are more heterogeneous than in the SSC-I.

The performance of the CPs’ annotation solutions has improved against the SSC-II in comparison to the SSC-I: the precision against the SSC-II has increased in comparison to the SSC-I. Recall has also improved. This result shows that the SSC-II incorporates characteristic features that are shared amongst all annotation solutions.

In the SSC-II the annotation solutions of the PPs for chemical entities show lower performance in comparison to the SSC-I (refer to fig. 2). The performance of the CPs’ annotation solutions has improved. Altogether the distribution of the performances of the PPs’ annotation solutions and the CPs’ solutions is comparable.

**Figure 5:**
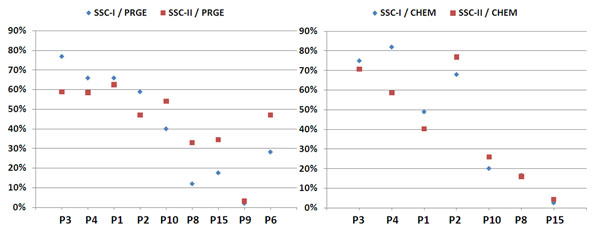
**(F-measure performances for PRGE and CHED):** The left and the right diagram show the performances for the different annotation solutions against the SSC-I (blue diamond) and against the SSC-II (red box). Each pair-wise entry represents a single annotation solution. The first four solutions have been provided by the PPs, the other solutions are taggers from the CPs. The left diagram shows the results for the PRGE annotations and the right diagram shows the results for the CHED annotations.

As can be seen in fig. 5, the performances of the annotation solutions for the four PPs deteriorated when comparing the performance against SSC-II instead of SSC-I. Furthermore, the performance of the four PPs against the SSC-II shows an F-measure that seems to be more evenly distributed across the different PPs, i.e. the systems seem to be more equal.

The performances of the CPs’ annotation solutions have improved when moving from the SSC-I to the SSC-II. This result can be explained by the fact that the contributions of the CPs have been included into the SSC-II in comparison to the SSC-I.

The results from the comparison of the annotation solutions for the chemical entities are not as clear as the results for the annotation of proteins/genes. In the case of the chemical entities, the performances of the PPs’ solutions deteriorate except for one PP. The performance of the CPs’ solutions varies to a small extent.

### Tagging of diseases and species measured against the SSC-II

**Figure 6:**
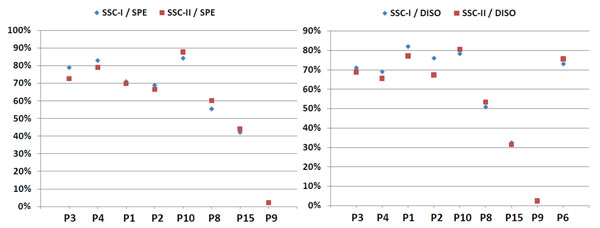
**(F-measure Performances for SPE and DISO):** The left diagram shows the results for the species annotations and the right diagram shows the results for the disease annotations (for details please refer to fig. 5).

The PPs’ annotation solutions and the CPs’ solutions show similar performance against the SSC-II and the SSC-I (ref. to fig. 6). The two corpora seem to have the same characteristics concerning the annotated entities in the corpus. In other words, the contribution of the CPs to the harmonised corpus did not change the quality of the silver standard corpus when producing the SSC-II from the PPs’ and the CPs’ contributions in comparison to the SSC-I. We can conclude that the annotation of disease entities is better normalised than the two other semantic groups, i.e. chemical entities and protein/genes, respectively.

Similar to the assessment of disease annotations, the species tagging solutions of the PPs and the project CPs did not vary when the annotations were evaluated against the SSC-II in comparison to the SSC-I. For both corpora, the annotation solutions yielded similar results. This leads to the conclusion that the SSC-I and the SSC-II have similar annotations and also to the result that the different contributing systems had similar performances right from the beginning. Overall, we can conclude that the representation of species is better normalised or standardised in the scientific literature than chemical entities or gene/protein representations.

In the last analysis, we compared the F-measures reached from the individual systems against the SSC-II directly against the F-measures from the SSC-I (ref. to table 5). This should give an overview on the solutions that gained performance in the SSC-II over the SSC-I and the other solutions that deteriorated their performance. When analysing the performance of the different solutions for species annotations and for diseases annotations, we find only small differences in the performances of the systems against the SSC-I and the SSC-II.

**Table 5:** The table shows the direct measurement of the SSC-I against the SSC-II that has been generated with the similarity measure of 98% cosine similarity scoring and a 3-vote agreement between the participants. The comparison is based on a 98% cosine similarity score.

	Reference SSC-I (cos 0.98)			
SSC-II	DISO	SPE	PRGE	CHED
Rec	89.0%	94.5%	59.7%	49.6%
Prec	71.6%	90.0%	96.8%	49.4%

F-meas	79.3%	92.2%	73.8%	49.5%

### Direct measurement of the SSC-I against the SSC-II

In the direct comparison between the SSC-I and the SSC-II, the annotations for SPE and DISO show better agreement than the comparison of the annotations for PRGE and CHED. The latter shows the lowest performance indicating that higher diversity exists between the two corpora.

## Discussion & conclusions

### Manual inspection of the SSC-I and the SSC-II

The manual analysis of the SSC-I and the SSC-II is ongoing work. Due to the size of the corpus, it requires special IT solutions to oversee the regularities and irregularities in the corpus. A selection of irregularities result from the methods applied. First, a number of annotations are not captured (“false negatives”, FN, reduced recall) if none of the solutions identifies the entities. An increasing number of contributing annotation solutions reduces the risk that annotations are missed: a bigger number of included annotation solutions lead to a bigger number of annotations that are captured. This achievement is counterbalanced by the number of agreements that have to be available at minimum to accept an annotation.

Second, for the same type of entity, e.g. “insulin”, different annotation solutions use a different tag, e.g. PRGE instead of CHED and vice versa. The harmonisation of the corpus can account for this, but will not produce this type of polysemous annotation throughout the whole corpus, since not all mentions have been consistently annotated with the two different groups over the whole corpus.

Third, inflections of terms, e.g. “tumour” vs. “tumours” and “bear” vs. “bearing”, lead to disagreements between the different annotation solutions. In the first case, the inflectional variability could be resolved and would lead to higher agreement, in the second case assumptions about the usage of the verb or noun have to be made to resolve conflicts.

## Conclusions

The comparison of the proposed solutions against the SSC-I is a new approach to evaluate annotation solutions. Until now, no large-scale corpus was available to achieve this task. In addition, it became clear that the SSC-I is homogeneous enough to be used as training data to achieve the same annotation task across the different semantic groups.

The generation of a harmonised corpus is a challenging task, but the presented results demonstrate that the produced harmonised corpus integrates the characteristics from the different annotation solutions. As a result, we can determine the features in the harmonised corpus by the annotation solutions that contribute to the generation of the SSC.

From a different perspective, we can argue that each of the used annotation solutions represents a piece of the complete annotation task. The more solutions are combined, the more closely we approximate an assumed consensus in the annotation task, which can be reproduced with a machine-learning tagging solution.

## Competing interests

The authors declare that they have no competing interests.

## Authors' contributions

DRS and AJY have prepared the manuscript. AJY, CL, SK, and IL have prepared annotated corpora and contributed to the analysis of the PPs and CPs annotations. NK, PC, EkB, and ElB have prepared annotated corpora. KH, DM, EvM, JK, and UH are part of the challenge planning and execution team. The remaining authors have contributed annotated corpora as challenge participants. All authors have revised the manuscript and the results.
